# Epidemiology of emergency ambulance service calls related to COVID-19 in Scotland: a national record linkage study

**DOI:** 10.1186/s13049-022-00995-6

**Published:** 2022-01-28

**Authors:** David Fitzpatrick, Edward A. S. Duncan, Matthew Moore, Catherine Best, Federico Andreis, Martin Esposito, Richard Dobbie, Alasdair R. Corfield, David J. Lowe

**Affiliations:** 1 Scottish Ambulance Service, Education and Professional Development Department, Grangemouth Road, Falkirk, Fk2 9AA UK; 2 Faculty of Health Sciences and Sport, Pathfoot Building, University of Stirling, Stirling, FK9 4LA UK; 3 Faculty of Social Science, University of Stirling, Stirling, FK9 4LA UK; 4 Nesta, Data Analytics Practice, The Bayes Centre 47, Potterrow, Edinburgh, EH8 9BT UK; 5 Scottish Ambulance Service Clinical Directorate, 1 South Gyle Crescent, Edinburgh, EH12 9EB UK; 6 Public Health Scotland, Gyle Square, 1 South Gyle Cresent, Edinburgh, EH12 9EB UK; 7 Emergency Department, Royal Alexandra Hospital, Paisley, PA2 9PN UK; 8 Emergency Department, Queen Elizabeth University Hospital, Glasgow, G51 4TF UK

## Abstract

**Background:**

COVID-19 has overwhelmed health services across the world; its global death toll has exceeded 5.3 million and continues to grow. There have been almost 15 million cases of COVID-19 in the UK. The need for rapid accurate identification, appropriate clinical care and decision making, remains a priority for UK ambulance service. To support identification and conveyance decisions of patients presenting with COVID-19 symptoms the Scottish Ambulance Service introduced the revised Medical Priority Dispatch System Protocol 36, enhanced physician led decision support and prehospital clinical guidelines. This study aimed to characterise the impact of these changes on the pathways and outcomes of people attended by the SAS) with potential COVID-19.

**Methods:**

A retrospective record linkage cohort study using National Data collected from NHS Scotland over a 5 month period (April–August 2020).

**Results:**

The SAS responded to 214,082 emergency calls during the study time period. The positive predictive value of the Protocol 36 to identify potentially COVID-19 positive patients was low (17%). Approximately 60% of those identified by Protocol 36 as potentially COVID-19 positive were conveyed. The relationship between conveyance and mortality differed between Protocol 36 Covid-19 positive calls and those that were not. In those identified by Protocol 36 as Covid-19 negative, 30 day mortality was higher in those not conveyed (not conveyed 9.2%; conveyed 6.6%) but in the Protocol 36 Covid-19 positive calls, mortality was higher in those conveyed (not conveyed 4.3% conveyed 8.8%). Thirty-day mortality rates of those with COVID-19 diagnosed through virology was between 28.8 and 30.2%.

**Conclusion:**

The low positive predictive value (17%) of Protocol 36 in identifying potential COVID-19 in patients emphasises the importance of ambulance clinicians approaching each call as involving COVID-19, reinforcing the importance of adhering to existing policy and continued use of PPE at all calls. The non-conveyance rate of people that were categorised as COVID-19 negative was higher than in the preceding year in the same service. The reasons for the higher rates of non-conveyance and the relationship between non conveyance rates and death at 3 and 30 days post index call are unknown and would benefit from further study.

**Supplementary Information:**

The online version contains supplementary material available at 10.1186/s13049-022-00995-6.

## Introduction

COVID-19 has overwhelmed health services across the world; its global death toll has exceeded 5.3 million and continues to grow [1]. There have been over 15million cases of COVID-19 in the UK, with over 1 million  of these occurring in Scotland, which of the four UK nations, currently has the third highest death rate at 177.6 per 100,000 population [2]. While the vaccination programme appears to have eased the immediate pressures within the UK, the evolution of new variants, and clinical presentations, pose a constant threat [3, 4]. Therefore, the need for rapid and accurate identification, enhanced by proactive approaches to the development of appropriate care and decision making, remains a priority.

Ensuring timely allocation of ambulances and subsequent conveyance decisions based on clinical acuity has been a longstanding challenge for ambulance services [5]. These challenges have been amplified during COVID-19, when ambulance services have found themselves under periods of intense pressure whilst trying to match resource with demand [6]. It is unsurprising, therefore, that the UK Association of Ambulance Chief Executives have stated that COVID-19 is the biggest challenge their services have ever faced [7]. The pace at which COVID-19 spread necessitated rapid development and implementation of clinical guidance. Subsequently, clinical guidance for the prehospital care of people with suspected COVID-19 was informed by constantly evolving evidence from in-hospital settings and was continuously refined via expert concensus. [8]

The ambulance care system in Scotland often begins with a 999 call to an emergency call handler based in one of three ambulance control centres. Consistent with many services in the UK and internationally, Scottish Ambulance Service (SAS) call handlers use the Medical Priority Dispatch System (MPDS) to triage the patient using one of 36 protocols and dispatch an appropriate resource [9]. Three key components were introduced to support pre-hospital care of COVID-19 in Scotland. Firstly, the MPDS initiated a modified protocol (Protocol 36) to include specific COVID-19 symptom-focused questions (AEDR, 2020) [10]. Protocol 36, originally developed for the SARS pandemic in 2003, was adapted to the current pandemic to (i) facilitate early, accurate identification of patients presenting with COVID_19 symptoms, (ii) support an appropriate ambulance response, and (iii) to identify geographical COVID-19 variance thereby informing ambulance, hospital, community and public health responses [10]. Secondly, Covid ‘hubs’ were introduced in each major Health Board area to enable professional-professional discussions. They were staffed by a range of experienced clinicians/physicians who provided additional, remote senior clinical decision support for ambulance clinicians (Paramedics, Technicians and Advanced Paramedics/Clinical Advisors [based in ambulance control]) and were aimed at ensuring the most appropriate pathway for possible COVID-19 patients. Thirdly, a clinical acuity guideline (informed by Scottish Government guidelines and evolving science) was introduced. This provided support for clinicians to identify COVID-19 symptoms and those higher risk patients who would benefit from further professional-professional discussion with a senior physician based in the Covid hub. These discussions between ambulance clinicians, physicians, patients and relatives would ultimately lead to shared decisions on conveyance.

The aim of this study was to describe and summarise the characteristics of people identified by ambulance service telephone triage as having COVID-19 symptoms during the first wave of COVID-19. Hereafter referred to as ‘Protocol 36 COVID-19 + ve’. Patient demographics and clinical acuity using the National Early Warning Score (NEWS) will be presented along with MPDS Protocol 36 performance, Patient Pathways and mortality rates. It is anticipated these data will enhance understanding of this patient population and improve future COVID-19 pre-hospital patient identification and management.

## Methods

### Study design and data

The study employed a retrospective cohort design using pseudonymised linked data from the NHS Public Health Scotland Unscheduled Care Data Mart [11]; specifically the Electronic Research and Data Innovation Service (eDRIS) COVID-19 Research Database [12]. This provided person level linked-data using established probabilistic and deterministic matching techniques, based on unique identifiers including NHS number, name and date of birth.

The following data sources were used:

- Scottish Ambulance Service patient data
- Emergency Departments (A&E2)
- NHS24
- GP Out-of-Hours
- General inpatient and day case hospital admission episodes (Scottish Morbidity Records SMR01)
- Laboratory confirmed COVID-19 (Electronic Communication of Surveillance in Scotland Virology),
- Continuous Unscheduled Care Pathway (where contact with one service happens within 24 h of the previous service.
- Death records (National Records of Scotland).

Cases were identified as calls to the Scottish Ambulance Service coded by the MPDS Protocol 36, which indicates the presence of COVID-19 symptoms. It was possible for one call to the ambulance service to refer to more than one individual so the unit of analysis for this study is ‘person-call’. That is, each case is a unique person-emergency call combination.

### Study setting and population

The Scottish Ambulance Service (SAS) is a national service covering 30,420sqm and serves a population of over 5.4 million [13]. During 2019/20, the service responded to over 806,000 incidents of which over 540,000 were categorised as emergencies [14]. The service is primarily delivered by paramedics (n = 1707) and emergency medical technicians (n = 1563) who work across a mix of urban, remote and rural settings (P. Bowtle, personal communication, 13th April 2021).

This study includes data on patients attended by the ambulance service from 1st April 2020 to 31st August 2020. Using previously published call data [14] we anticipated approximately 225,000 incidents over the 5 month period. We report results for adults aged 16 or more years of age on first contact.

### Statistical analysis

Analyses were conducted in Public Health Scotland’s National Safe Haven adhering to current disclosure protocols [15]. Using these linked data we determined the (i) demographic and clinical characteristics (ii) predictive ability of MPDS Protocol 36 and (iii) described the pathways taken and outcomes related to conveyance decisions.

#### Demographic and clinical variables

We report descriptive statistics for age, gender, SIMD, and comorbidities by Protocol 36 flag status. Scottish Index of Multiple Deprivation (SIMD):/scottish-index-multiple-deprivation-2020/) was used to categorise patient-calls by SIMD quintile, in order to determine whether calls identified by MPDS Protocol 36 differed in terms of area-based socioeconomic profile to other calls attend by SAS during this time period.

Clinical variables reported: first recorded National Early Warning Score (NEWS) [16] and Chronic Conditions (Unscheduled Care Data Mart) [11]. We report descriptive statistics sub-grouped by combinations of MPDS Protocol 36 status, subsequent virology test and conveyance. Three and 30 day mortality rates are reported as outcomes.

Positive predictive ability of Protocol 36: The positive predictive value was calculated as the proportion of people identified by Protocol 36 as potentially having COVID-19 who subsequently receive a positive COVID-19 diagnosis confirmed by virology within 10 days of the call, among those receiving virology testing.

Patient Pathways and Mortality analysis: Patient pathways describe the subsequent service use for people attended by the ambulance service. A patient pathway may contain more than one call to the ambulance service. The pathway analysis begins at the first ambulance service attendance (the index call) from 1 April 2020. We report continuous care pathways which are episodes of care where each care episode occurs within 24 h of the previous one.

The association between Protocol 36 COVID-19 + ve status, conveyance and mortality at 30 days was assessed using logistic regression. The difference between the effect of conveyance on mortality between Protocol 36 COVID-19 + ve cases and other cases was assessed using an interaction term in the regression. Analyses were adjusted for gender and age group.

Patient and public involvement: The timescale of the rapid grant funding process precluded patient and public involvement in setting the research question, in the design or implementation of the study.

## Results

### Patient demographics with COVID-19 test status

The ambulance service responded to 214,082 patient calls during the five-month study period where Protocol 36 was used. Of these the ambulance call handlers, using Protocol 36, categorised 3.4% (n = 7,305) as Protocol 36 COVID-19 + ve. Table 1 illustrates that the sociodemographic profiles of people classified by Protocol 36 as COVID-19 + ve do not differ from the overall profile of people seen in terms of age, gender and having two or more co-morbidities. Socio economic status were very similar across SIMD quintiles (these data are included as Additional file 1.

**Table 1:** Demographic characteristics and proportions of patients attended by Protocol 36 COVID-19 status

	Protocol 36 COVID-19 status	
Demographics	Positive	Negative
N patient/calls	7305	206,777
Median age in years(Interquartile range)	66 (50–78)	66 (47–80)
% Female	49.75	48.97
% with 2 + co-morbidities	58.07	60.26

### Clinical characteristics and mortality rates of patient-calls sub-grouped by Protocol 36 COVID-19 status and subsequent virology test status

Table 2 shows the clinical characteristics and mortality rates for patient-calls sub-grouped by Protocol 36 COVID-19 + ve status and subsequent virology test status.

**Table 2:** Characteristics of patient-calls subgrouped by MPDS Protocol 36 COVID-19 status and virology test status

Subgroup	N patient/calls	Median age (years) (IQR)	% Female	Median NEWS* (IQR)	% with 2+ co-morbidities	30 day mortality rate	Conveyed %
Missing data rates (% rounded)	N/A	N/A	N/A	12.5	N/A	N/A	N/A
Protocol 36 COVID-19 negative/no test evidence	142,985	60 (35)	51	1 (3)	57	6.4	63
Protocol 36 COVID-19 negative/tested negative	61,013	75 (22)	52	2 (4)	68	8.6	88
Protocol 36 COVID-19 positive/no test evidence	4,737	62 (31)	52	1 (3)	55	4.4	48
Protocol 36 COVID-19 positive/tested positive	438	70.5 (23)	43	6 (6)	58	28.8	80
Protocol 36 COVID-19 negative/tested positive	2779	75 (24)	48	5 (6)	66	29.0	83
Protocol 36 COVID-19 positive/tested negative	2,130	72 (24)	49	3 (6)	66	8.7	82

*National Early Warning Score

### Predictive value of MPDS Protocol 36 identification of potential COVID-19 calls

We calculated the positive predictive value of protocol 36 for identifying likely COVID-19 positive presentations. That is, we calculated the proportion of people suspected as having COVID-19 (Protocol 36 COVID-19 + ve) who were subsequently found to have COVID-19 by virology testing (Tested Positive). The positive predictive value was only calculated for those who received laboratory PCR confirmed SARS-COV-2 infection within 10 days of index call, as this is this gold standard to determine whether the Protocol 36 COVID-19 correctly identified COVID-19 cases. From 7,305 patients identified as potentially having COVID-19, 438 (6%) were subsequently identified by virology results as COVID-19 positive, 2130 had negative virology results. A significant proportion (65%, n = 4737) had no test results. The overall positive predictive value was 17.06% (95% CI 16.77–17.35). The positive and negative predictive values for each month between April and August 2020 are presented as additional material. The positive predictive value dropped from April 2020 through June 2020, however during this time the test positivity rate (that is the proportion of positive of COVID-19 test results amongst calls that are tested within 10 days of the initial ambulance call) also decreased. The PPV over the study duration is presented as Additional file  2.

### Patient pathways

**Fig. 1:**
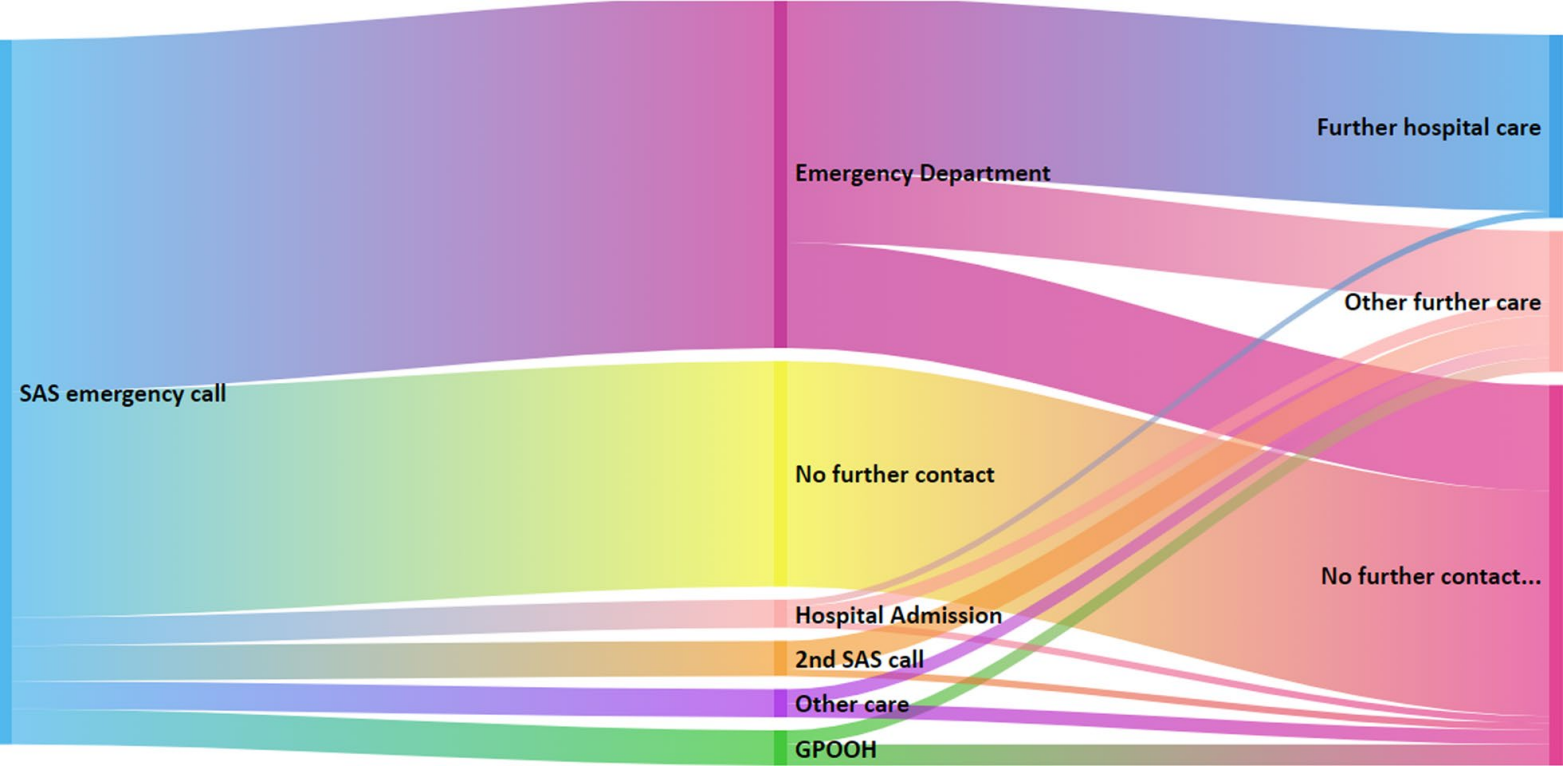
Healthcare events following initial ambulance service call where initial call was Protocol 36 COVID-19 positive. Key: *SAS Emergency Call* Call responded to by the Scottish Ambulance Service; *GPOOH* General Practitioner Out of Hours Service; *Other care* NHS 24, virology testing or death

For this section calls are organised into patient pathways. A pathway is the patient journey after an initial call to SAS. A pathway may contain more than one SAS call. There were 5720 patient pathways that began with a call attended by the ambulance service where COVID-19 was suspected (Protocol 36 COVID-19 + ve). For half of these (50%, n = 2860), their next unscheduled care episode was the Emergency Department. Smaller proportions were either admitted directly to hospital (4%, n = 229), used GP out of hours services (5%, n = 286) or were referred to alternative NHS services such as NHS24 or virology tests. In addition, where the next episode in the pathway is patient death, this is also included in this category (10%, n = 572). The rest remained at home and did not have any further care episodes within their unscheduled care pathway (32% n = 1830). Around half (n = 1430) of those taken to the Emergency Department had further hospital care (i.e. hospital admission) (Fig. 1).

### Conveyance decisions and Mortality at 30 days post ambulance call

The unit of analysis for the following section is the patient-call. Patient-calls that were identified as Protocol 36 COVID-19 + ve were less likely to be conveyed with 59.7% (4,358/7,305) conveyance rates, compared to those that were not identified as Protocol 36 COVID-19 + ve, where conveyance was 70.8% (146,303/206,777) (see Table 3).

**Table 3:** Relationship between conveyance and death within 30 days by Protocol 36 COVID-19 status

							Reason for call stopped				
	Protocol 36 COVID-19 status	N (%)	Median NEWS*	IQR NEWS	Death within 3 days (%)	Death within 30 days (%)	Refused (%)	Referred (%)	Advice (%)	See and treat (%)	Other (%)
Not conveyed	Protocol 36 COVID-19 negative	60,474 (29.2)	1	3	3,980 (6.6%)	5,567 (9.2)	11,973 (19.8)	8,345 (13.8)	18,263(30.2)	2,479 (4.1)	19,412(32.1)
	Protocol 36 COVID-19 positive	2,947 (40.3)	1	3	47 (1.6)	127 (4.3)	475 (16.1)	586 (19.9)	1,188 (40.3)	206 (7.0)	492 (16.7)
Conveyed	Protocol 36 COVID-19 negative	146,303 (70.8)	2	4	3,179 (2.2)	9,658 (6.6)					
	Protocol 36 COVID-19 positive	4,358 (59.7)	3	5	131 (3.0)	384 (8.8)					

*National Early Warning Score

Logistic regression results confirm that for the majority of calls, people who were conveyed, were less likely to die within 30 days than those who were not conveyed (OR 0.64 95% CI 0.62–0.67) after adjustment for age group and gender. The statistically significant interaction effect between Protocol 36 COVID-19 + ve categorisation by the ambulance control and conveyance to hospital (OR 3.06 95% CI 2.48–3.78) indicates that the effect of conveyance on odds of death within 30 days is 3 times greater for those categorised as Protocol 36 COVID-19 + ve than those patient calls who were not categorised as Protocol 36 COVID-19 + ve after adjustment for gender and age category. As might be expected, the older age groups had higher odds of 30 day mortality compared to those under 30 (e.g. those over 90 have 20 times greater odds of mortality within 30 days compared to the reference group of those between 16 and 30 years old after adjustment for covariates). Females have lower odds of 30 day mortality than males (OR 0.68 0.66–0.71 conditional on covariates). Other potential interactions with conveyance (gender, age group, SIMD were tested and found to be not significant) (Table 4).

**Table 4:** Logistic regression of Protocol 36 COVID-19 status on death within 30 days

	Odds Ratio	p	95% CI
Protocol 36 COVID-19 + ve	0.43	< 0.001	0.36–0.52
Conveyance	0.64	< 0.001	0.62–0.67
Protocol 36 COVID-19 + ve/conveyance interaction	3.06	< 0.001	2.48–3.78
Age category 30	3.25	< 0.001	2.76–3.83
Age category 50	7.74	< 0.001	6.63–9.05
Age category 70	14.28	< 0.001	12.24–16.65
Age category 90	20.66	< 0.001	17.61–24.24
Gender (female ref male)	0.68	< 0.001	0.66–0.71

## Discussion

The 214,000 calls attended during the study period were less than the 225,000 calls that had been anticipated based on calls during the same time-period in the preceding year. The demographic and available clinical characteristics of those people flagged as Protocol 36 + ve were very similar to the general population of people attended by the ambulance service. Those with positive virology requiring ambulance assistance had notably high 30-day mortality rates (between 28.8 and 30.2%). Some differences were noted; those with a COVID-19 + ve virology presented with higher NEWS scores than other groups (median 5 and 6) irrespective of whether identified by MPDS Protocol 36. Protocol 36 identified 7,305 people (3.4%) as potentially having COVID-19. But, the positive predictive value of this protocol was low (17%). Sixty percent of calls identified as Protocol 36 COVID-19 + ve resulted conveyance whereas conveyance rates were much higher in Protocol 36 COVID-19 − ve cases (70.8%). Among Protocol 36 + ve cases, mortality rates were higher in the group of patients conveyed for further care. But highest 30 day mortality rates were in those who were Protocol 36 − ve and not conveyed (9.2%).

### Triage systems

During the early stages of COVID-19 the Medical Priority Dispatch System Protocol 36 was modified to support the COVID-19 outbreak along with a bespoke training package for call handlers to “*help identify and manage suspected infected patients in a manner that utilizes scarce EMS, hospital, and community resources effectively and efficiently during a declared pandemic*” [10], (pg.2). During the pandemic Protocol 36 replaced Chief Complaint Protocols ‘breathing problems (06)’, ‘chest pain (10) and ‘sick person with flu like illness (26)’ to support appropriate triage of those with COVID-19 symptoms. Some data suggests that this protocol has eased pressures on control systems and operations as it asks key questions to differentiate between, for example, chest pain likely to be associated with COVID-19 from chest pain of cardiac origin [18]. But, the true impact on patient outcomes of any of such adjustments remain unknown and further study in this area, using high quality data sources, is urgently required. During August 2020, the final month of our study, the MPDS reverted to using the standard Chief Complaint protocols (as outlined previously), however, this did not dramatically impact on its PPV (Additional file 2: Table 2), with Protocol 36 still applied to those patient reporting COVID-19 symptoms as their chief complaint. To investigate the impact of adjustments to Protocol 36 was beyond the aims of this study but does warrant further investigation.

Previous studies have demonstrated MPDS’s predictive ability to vary considerably for high acuity conditions/presentations such as Cardiac Arrest (PPV 27–67%, Stroke (PPV 9.4–47%) and Major Trauma (PPV 45.3%). [17] And, its predictive ability across many other chief complaints is moderate to low [17]. Similarly, our study determined that the predictive ability of Protocol 36 was low at 17%. However, this was slightly lower than in two other recently published, although smaller, studies. The first, a study by Pineo et al., [19] examined the positive predictive value of MPDS Protocol 36 in New York City, USA and determined this to be 24.3% and the second by Spangler et al., [20] a nurse led triage system in Sweden, reported a PPV of 24.7% (in tested patients). It is not known why there is a difference in PPV using  the same or similar systems. Contextually, Ambulance Control Centres remain under considerable pressure, and any additional questions during triage place further workload on the system. While additional protocols may introduce a benefit to patients, our results indicate that, within the context of its stated aims, Protocol 36 has little utility and, therefore, would benefit for further refinement. There is a balance to strike between additional workload and gain. Our study, along with those by both Pineo et al [19] and Spangler et al., [20] highlight the significant challenges services face in identifying COVID-19 using telephone and clinical triage. Importantly, these poor PPV during triage reinforce current NHS recommended enhanced PPE approaches during prehospital patient care.

### Relationship between conveyance, clinical acuity and outcomes

Non-conveyance outcomes are essential measures of a pre-hospital system’s safety and effectiveness. Non-conveyance figures vary considerably across UK services [21]. During the study period clinical guidelines changed rapidly based on the evolving scientific evidence and expert opinion [6, 8]. Thresholds for professional-to professional discussion based on key clinical variables and co-morbidities were modified throughout this period; measuring the impact of these modifications was beyond the scope of this study. Overall, 29.2% of patients who were Protocol 36 − ve remained at home after ambulance clinician assessment. Although this non-conveyance rate is considerably higher than the circa 19% non-conveyance rate published for the same service [14] in the preceding year definitions of ‘non-conveyance’ can vary and this may limit direct comparison. Higher non-conveyance rates were present in Protocol 36 + ve patients, where rates reached almost 40%, this being slightly higher than the 35–38% historical non-conveyance rates across English ambulance services between 2013 and 2016 [21]. Notably, we found that the relationship between conveyance and mortality differed by Protocol 36 status. In people who were Protocol 36 − ve, non-conveyance vs conveyance was associated with an increased risk of mortality (9.3% vs 7.0%). Whereas for Protocol 36 + ve calls, conveyance was associated with an increased risk of mortality (8.8% vs 4.3%).

Our study was not undertaken to explain differences in mortality rates between the Protocol 36 − ve non-conveyed group and other groups. However, it is useful to explore data from other sources as potential explanations. Crude mortality rates from Scottish hospital discharge data [22] demonstrate increases in 30 day mortality from 3.1 and 4.0% (2014–2019) to 6.1% during our study period [23]. These are still lower than the ambulance 9.3% non-conveyance 30 day mortality rates. Few studies have investigated and reported ambulance population mortality rates but Christensen et al. [24] reported an average 30 day mortality rate of 4.7%. Again our mortality rates are higher than hospital discharge data and past data on prehospital studies.

There are numerous variables that will have impacted on mortality during our study period. Those with COVID-19 positive virology had higher median ages and NEWS than other groups and as many were not identified during telephone triage they would fall into our Protocol 36 − ve non-conveyed group; mortality rates in those with COVID-19 + ve virology ranged between 28.8 and 30.2%. Advancing age also increased the odds of dying at 30 days (28.2–30.2%) and higher NEWS scores have previously been demonstrated as a pre-hospital predictor of mortality [25]; both of which were higher in the COVID-19 + ve virology groups. Nursing home deaths accounted for 56.7% (n = 2261) of all COVID-19 related deaths recorded during our study period [26]. It is likely that many of those individuals will have had contact with ambulance services and subsequently not conveyed after discussions with patients, relatives and senior clinicians.

With respect to patient health behaviours we know patients health seeking behaviour changed with patient willingness to attend Emergency Departments or seek help from Primary Care affected [27–29]. Emergency Department attendances fell 40% as did ambulance call-outs [27]. Qualitative findings suggest that some of those with high acuity, life-threatening conditions either did not seek care or received suboptimal assessment leading to delays in definitive interventions [27]. Reportedly some were fearful of contracting COVID-19 in hospital thus impacting on normal health-seeking behaviours. Our data has differencing refusal rates between Protocol 36 + ve status; those with Protocol 36 − ve having higher refusal rates. However, understanding the reasons, appropriateness and safety of refusal vs see and treat or referral is challenging and so recorded reasons for non-conveyance should be interpreted  with caution [30]. The impact of COVID-19 on clinical decision making of ambulance clinicians during the study period has been investigated by the authors in a separate qualitative study [31].

Overall, understanding the impact on patient outcomes of NHS instigated virus spread reduction measures is challenging and beyond the scope of this study. It is likely a factor of the complex interactions between patient demographics, clinical presentation/acuity, pre-existing morbidity and health behaviours influenced by the pandemic.

### Strengths and limitations

This study has various strengths and limitations. As the data have national coverage, there is no inherent selection bias. To our knowledge, this study is one of the first analysis of national level ambulance service data of clinical practice during the COVID-19 pandemic to include clinical variables that enable clinical acuity comparisons between patient groups. Our study only included people aged 16 years or over. In common with other data linkage studies [6],^,^ [30] this study experienced problems with data quality which limited some of the analyses that could be undertaken. However, data were complete for the reported outcomes. The assessment of the positive predictive value for the MPDS Protocol 36 is limited by the fact only 35% of calls were linked to virology test results. Virology testing was predominantly available for people who were conveyed to ED.

### Clinical and policy implications

The use of subjective and generic clinical presentations of the COVID-19 virus in Protocol 36 does not support the accurate identification of COVID-19 in patients presenting to the ambulance service population. Inaccurate data could impact on essential clinical and community resource management decisions and we therefore suggest its utility in its current format is limited. Our data and findings are generalizable to other EMS systems that operate within interlinked public health systems using MPDS. There were some indications that PPV was increasing slightly from June to August but there are too many unknowns to speculate reasons for this. Nevertheless, this may point towards the potential to develop and test a refined Protocol 36.

### Further research

Factors such as risk of COVID-19 infection from hospital attendance may be impacting on clinician and patient decision making about conveyance and merit further investigation. There was a higher rate of death at 3 and 30 days in the group that were Protocol 36 − ve and were not conveyed to hospital. The reasons for this are unknown and could relate, at least in part, to clinically appropriate decision making (eg. non-conveyance of patients with palliative or end of life care from a nursing home). Further investigation of the reasons for non-conveyance of patients during the COVID-19 pandemic could enable improved guidance and support for ambulance clinicians and people receiving pre-hospital emergency care. Further analysis of clinical data is also required to determine clinical predictors of hospital admission and outcomes.

## Conclusions

The Scottish Ambulance Service dealt with 214,082 patient calls during the five-month study period. Non-conveyance rates and outcomes are a marker of the safety and effectiveness of EMS systems. The non-conveyance rates among people that were not categorised as Protocol 36 COVID-19 + ve were higher than in the preceding year. The reasons for the higher rates of non-conveyance and the relationship between non conveyance rates and death at 3 and 30 days post index call are unknown. This and would benefit from further study to enhance our understanding and support the development of effective decision support tools and ongoing education of ambulance clinicians. The MPDS protocol 36 used by the service to identify individuals who potentially had COVID-19 had very poor ability to predict which individuals had COVID-19 and which did not. This finding suggests that ambulance clinicians should treat each call during the pandemic as potentially involving an individual who is COVID-19 + ve.

## Supplementary Information

**Additional file 1.** Protocol 36 COVID-19 status by SIMD quintile.

**Additional file 2.** Predictive value of Protocol 36 of COVID-19 status across the 5 month study period.

## Data Availability

The dataset analysed for this study is available on application from NHS Public Health Scotland: ISD Services|Electronic Data Research and Innovation Service (eDRIS)|COVID-19|ISD Scotland.
