# Supplementary material for: Epidemiology of emergency ambulance service calls related to COVID-19 in Scotland: a national record linkage study
Source: Scand J Trauma Resusc Emerg Med. 2022 Jan 28;30:9. doi: 10.1186/s13049-022-00995-6 (PMC8795941; doi:10.1186/s13049-022-00995-6)
Supplement: Supplementary file 1 — Additional file 1. Protocol 36 COVID-19 status by SIMD quintile. [file 13049_2022_995_MOESM1_ESM.docx]

*Additional Material Tables 1*

*Socioeconomic Status*

Around 30% of all calls came from geographical areas with the highest levels of socioeconomic disadvantage. Only 11.5% of calls came from geographical areas with the lowest levels of sociodemographic disadvantage. The proportion of calls from each quintile of area-level geographic deprivation was very similar for calls that were Protocol 36 COVID-19 negative and those that were positive indicting that there was no socioeconomic patterning in the application of the Protocol as shown in Table 2.

| ***Table 1: Protocol 36 COVID-19 status by SIMD quintile*** | | |
| --- | --- | --- |
| Subgroup | Protocol 36 COVID-19  Negative | Protocol 36 COVID-19 Positive |
| 1 – Most Deprived | 30.49 | 31.15 |
| 2 | 23.91 | 24.92 |
| 3 | 19.32 | 18.38 |
| 4 | 14.84 | 13.89 |
| 5 – Least Deprived | 11.43 | 11.65 |
