# Supplementary material for: Epidemiology of emergency ambulance service calls related to COVID-19 in Scotland: a national record linkage study
Source: Scand J Trauma Resusc Emerg Med. 2022 Jan 28;30:9. doi: 10.1186/s13049-022-00995-6 (PMC8795941; doi:10.1186/s13049-022-00995-6)
Supplement: Supplementary file 2 — Additional file 2. Predictive value of Protocol 36 of COVID-19 status across the 5 month study period. [file 13049_2022_995_MOESM2_ESM.docx]

| ***Additional material table 2: Predictive value of Protocol 36 of COVID-19 status across the 5 month study period*** | | | | |
| --- | --- | --- | --- | --- |
| Group | Positive predictive value | Negative predictive value | Test positivity rate | N tests |
| Overall | 17.06 | 95.64 | 4.9 | 66,362 |
| April | 34.09 | 81.28 | 20 | 12,593 |
| May | 18.35 | 95.16 | 3.6 | 15,692 |
| June | 0.82 | 99.47 | 0.54 | 13,984 |
| July | 1.02 | 99.87 | 0.15 | 12,944 |
| August | 10.29 | 99.68 | 0.39 | 11,149 |
